# Characterizing the neuroimmune environment of offspring in a novel model of maternal allergic asthma and particulate matter exposure

**DOI:** 10.1186/s12974-023-02930-7

**Published:** 2023-11-02

**Authors:** Juan M. Tamayo, Hadley C. Osman, Jared J. Schwartzer, Kent E. Pinkerton, Paul Ashwood

**Affiliations:** 1 Department of Medical Microbiology and Immunology, and the M.I.N.D. Institute, University of California at Davis, 2805, 50th Street Sacramento, Davis, CA 95817 USA; 2 Program in Neuroscience and Behavior, Department of Psychology and Education, Mount Holyoke College, 50 College Street, South Hadley, MA 01075 USA; 3 Center for Health and the Environment, University of California at Davis, Davis, CA 95616 USA

**Keywords:** Neurodevelopment, Cytokines, Autism spectrum disorder (ASD), Schizophrenia, Neuroinflammation, Asthma/allergy, Fetal brain, Maternal immune activation (MIA), Maternal asthma and allergy (MAA)

## Abstract

Inflammation during pregnancy is associated with an increased risk for neurodevelopmental disorders (NDD). Increased gestational inflammation can be a result of an immune condition/disease, exposure to infection, and/or environmental factors. Epidemiology studies suggest that cases of NDD are on the rise. Similarly, rates of asthma are increasing, and the presence of maternal asthma during pregnancy increases the likelihood of a child being later diagnosed with NDD such as autism spectrum disorders (ASD). Particulate matter (PM), via air pollution, is an environmental factor known to worsen the symptoms of asthma, but also, PM has been associated with increased risk of neuropsychiatric disorders. Despite the links between asthma and PM with neuropsychiatric disorders, there is a lack of laboratory models investigating combined prenatal exposure to asthma and PM on offspring neurodevelopment. Thus, we developed a novel mouse model that combines exposure to maternal allergic asthma (MAA) and ultrafine iron-soot (UIS), a common component of PM. In the current study, female BALB/c mice were sensitized for allergic asthma with ovalbumin (OVA) prior to pregnancy. Following mating and beginning on gestational day 2 (GD2), dams were exposed to either aerosolized OVA to induce allergic asthma or phosphate buffered saline (PBS) for 1 h. Following the 1-h exposure, pregnant females were then exposed to UIS with a size distribution of 55 to 169 nm at an average concentration of 176 ± 45 μg/m^3^) (SD), or clean air for 4 h, over 8 exposure sessions. Offspring brains were collected at postnatal days (P)15 and (P)35. Cortices and hippocampal regions were then isolated and assessed for changes in cytokines using a Luminex bead-based multiplex assay. Analyses identified changes in many cytokines across treatment groups at both timepoints in the cortex, including interleukin-1 beta (IL-1β), and IL-17, which remained elevated from P15 to P35 in all treatment conditions compared to controls. There was a suppressive effect of the combined MAA plus UIS on the anti-inflammatory cytokine IL-10. Potentially shifting the cytokine balance towards more neuroinflammation. In the hippocampus at P15, elevations in cytokines were also identified across the treatment groups, namely IL-7. The combination of MAA and UIS exposure (MAA–UIS) during pregnancy resulted in an increase in microglia density in the hippocampus of offspring, as identified by IBA-1 staining. Together, these data indicate that exposure to MAA, UIS, and MAA–UIS result in changes in the neuroimmune environment of offspring that persist into adulthood.

**Supplementary Information:**

The online version contains supplementary material available at 10.1186/s12974-023-02930-7.

## Background

Neurodevelopmental disorders (NDD) are a broad group of disorders where central nervous system (CNS) development and function is altered [27]. NDD, including autism spectrum disorder (ASD) and attention deficit hyperactivity disorder (ADHD), are increasing in prevalence, with 1 in 6 children in the US experiencing a NDD in 2016 [125]. While there are some genetic origins of NDD, environmental factors such as exposure to pollution and maternal health are also recognized as risk factors [46, 100]. Mounting clinical and epidemiological data underscore the notion that environmental factors play a large role in both the underlying development of NDD and their impact on the severity of individual behavioral characteristics [52, 94, 99, 105, 114, 118, 120]. Pregnancy, in particular, marks a critical period when environmental insults have long-lasting effects on neurodevelopment.

The maternal immune system represents a key biological mechanism that links environmental factors to specific neurodevelopmental changes and later NDD diagnosis. For example, clinical studies have linked hospitalizations for bacterial and viral infections during pregnancy to an increased rate of birthing a child that will later be diagnosed with ASD [4, 14, 80, 116]. Moreover, laboratories have developed animal models that often use bacterial or viral mimics, such as lipopolysaccharide (LPS) or polyinosinic:polycytidylic acid (Poly(I:C)), respectively, to induce maternal inflammation during pregnancy and demonstrate a causal change in offspring brain and behavioral health [46]. Despite the efficacy of these models in demonstrating a link between maternal immune signaling and offspring neurodevelopment, they are limited in representing many of the common environmental sources of immune activation. With perhaps the exception of SARS-CoV-2, hospitalizations for viral infections are less frequent, and a recent meta-analysis of viral infections during pregnancy as a risk for children later being diagnosed with ASD was not supported [55]. Importantly, infections via bacterial and viral pathogens are only two of many ways in which the immune system can become activated, and researchers have begun to investigate other common environmental influences or immune conditions that can have a substantial impact on the maternal immune system and offspring brain development, notably allergic asthma.

Rates of asthma, like NDD, are currently increasing and represent a highly prevalent chronic disease that more commonly impacts ethnic/racial minority and socioeconomically disadvantaged groups in the US [23, 72]. Importantly, chances of asthma exacerbations increase during pregnancy, and the presence of asthma increases likelihood of birthing a child that is later diagnosed with ASD [1, 3, 25, 26, 33, 42, 47, 87, 88, 93]. Asthma is an inflammatory disease of the lungs characterized by bronchial hyperresponsiveness, persistent inflammation, airflow obstruction, and reduction of airflow [39, 96]. The allergens associated with asthma can vary and include, but are not limited to rodent allergen, cockroach allergen, pollen, and house dust mite [53, 72]. Given the incidence of allergies and asthma in the United States [10, 34] and the high prevalence of allergic triggers in urban environments [22, 64, 72, 106], there is pressing need to better understand the causal effects of asthma during pregnancy and the impact of specific allergic triggers on offspring neurodevelopment.

Using our mouse model of maternal allergic asthma (MAA) to initiate an immune response in pregnant mice, we have previously reported systemic elevations in interleukin-4 (IL-4) and IL-5 in dams [109, 115] that parallel clinical reports associating increased IL-4 and IL-5 in mid-pregnancy maternal serum samples of mothers with children later diagnosed with ASD [40]. Not only do the dams in our MAA model produce a similar allergic asthma cytokine profile to that observed in humans, but the offspring display characteristic ASD-like behaviors, such as decreased social interaction and increased repetitive-like behaviors [20, 109, 110]. Moreover, MAA produces transcriptional differences in microglia gene expression and neuroinflammation in both prenatal offspring and brain regions of adult mice [20, 115, 117]. These findings highlight the lasting changes that allergic asthma during pregnancy can have on offspring neurodevelopment. However, environmental insults associated with ASD can vary, and exposure to these environmental factors does not necessarily occur in isolation for humans. In fact, individuals often simultaneously encounter multiple inflammatory-inducing environmental stimulants, and less is known about the potential synergistic effects of these exposures on maternal immune response and subsequent offspring development [22, 64, 106].

Particulate matter (PM), via air pollution, is another environmental factor that is suspected to be associated with the risk of neuropsychiatric disorders/NDD such as schizophrenia, attention deficit hyperactive disorder (ADHD), and ASD [118–120]. Not only is PM linked to neurodevelopmental disruptions when exposures occur during pregnancy [21], but it has also been tied to exacerbated asthma responses and could potentially cause new onset cases of asthma [16, 17, 44, 108] although this remains controversial. PM is a complex mixture of constituents that contains organic compounds, soot, metals and metal-oxides, nitrates, and other elements in varying quantities depending on its source [113]. As such, it is necessary to characterize individual components of PM in order to effectively regulate air quality for limiting environmental exposure to toxicants.

Diesel exhaust is one common source of PM that has been identified as a risk factor for ASD [57, 103, 118–121]. Combustion-derived diesel exhaust includes soot particles containing elemental carbon and iron, which is the most common transition metal found in PM [127]. Iron in PM can occur from fuel additives and as a product of normal engine wear [74, 113], and iron-soot (IS) exposure has previously been shown to cause oxidative lung injury, induce inflammation of the lungs, and can cross the blood–brain barrier through nasal inhalation [48, 126, 127]. The links between PM and worsening of asthma symptoms and increased risk of neuropsychiatric disorders in offspring make PM exposure during pregnancy an important area that merits investigation.

There are many human studies and animal models investigating the links between gestational exposure to PM and neurodevelopmental outcomes in offspring [1, 2, 11, 118–120]. In addition, many studies have investigated the impacts of asthma as a risk factor for ASD, including our own [3, 20, 25, 26, 33, 42, 47, 68, 87, 88, 93, 109, 110, 115, 117]. However, these investigations consider asthma or PM only as independent risk factors for developing neuropsychiatric disorders. Despite the links between allergic asthma with ASD and the link between PM and ASD, there are no studies investigating the neuroimmune outcome on offspring of these environmental factors in conjunction. This is an apparent oversight given that PM exposure can worsen symptoms of asthma [44], and the source of environmental factors contributing to ASD are likely multifaceted.

In this study, we hypothesized that when mice are exposed to MAA and ultrafine iron-soot (UIS) particles during pregnancy (MAA–UIS), the neuroimmune outcomes will show heightened inflammation in offspring compared to that of MAA or UIS exposure alone. We also suspected that, because microglia can respond to changes in the neuroimmune environment through proliferation and are suspected to be associated with ASD behaviors [28, 51], we would see signs of functional differences in the frontal cortex and hippocampus—two brain regions implicated as being impacted developmentally in neuropsychiatric disorders such as ASD [6, 31, 51, 102]. Using our established mouse paradigm of maternal aerosol exposure to study offspring outcomes, we demonstrate that MAA and UIS alone, as well as MAA and UIS combined, alter the neuroimmune profile in the brains of offspring that is sustained from adolescence into early adulthood.

## Methods

### Animals

BALB/c male and female mice were obtained from breeding pairs originally purchased from Envigo Laboratories (Livermore, CA, USA) and maintained at University of California, Davis at the Center for Health and Environment, Davis, CA. Mice were housed with same-sex littermates and kept at ambient room temperature (23 °C) on a 12 h light/dark cycle (lights on at 0800 h) within ventilated cages with water and food provided ad libitum. All procedures were performed with approval by University of California Davis Institutional Animal Care and Use Committee and according to guidelines established by National Institute of Health Guide for the Care and Use of Laboratory Animals.

### Maternal allergic asthma and ultrafine soot particle exposure

Female BALB/c (*n* = 8/group) mice were sensitized with 10 µg of ovalbumin (OVA, Sigma, St. Louis, MO, USA) and 1 mg Al OH3 dissolved in 200 µl of phosphate buffered saline (PBS) injected intraperitoneally at 7 and 8 weeks of age, for a total of two injections pre-pregnancy. Control dams were injected with PBS alone. Dams were then mated with age-matched males the week after final sensitization, and checked daily for the presence of a seminal plug, which was noted as gestational day (GD) 0.5. Beginning on gestational day 2 (G2), dams were exposed to either aerosolized OVA to induce allergic asthma or phosphate buffered saline (PBS) for 1 h, depending on treatment group. Following the 1-h exposure, mice were placed in a 20 cm × 43 cm × 18 cm polycarbonate whole-body chamber for exposure to an aerosol of ultrafine iron-soot (target concentration of 200 µg/m^3^), including 40 µg/m^3^ of iron oxide nanoparticles, or sham control clean air (AIR). The total iron-soot generated was cooled and diluted with filtered air to achieve the desired concentration prior to reaching the exposure chambers. Mice were exposed for 4 h/day on G2, G4, G6, G9, G11, G13, G16, and G18 to PBS or OVA [MAA condition] and AIR or ultrafine iron-soot [UIS condition], resulting in a total of four treatment groups: PBS/AIR (PBS-AIR), MAA/AIR (MAA), PBS/UIS (UIS), MAA/UIS (MAA–UIS). The mass concentration of the soot concentration was found by weighing 25-mm Teflon-coated filters (Teflo, Pall, East Hills, NY) on a microbalance before and after sample collection. The average soot concentration over the exposure period was 176 ± 45 μg/m^3^ (SD). Following the last day of aerosol exposure (G18) pregnant mice were left undisturbed through parturition. Offspring were either sacrificed at postnatal day (P)15 or weaned at P21, housed with same-sex littermates, and sacrificed at P35 for brain tissue analysis.

### Generation and characterization of particles

UIS particles were generated as previously described by Hopkins et al. [48]. Briefly, particle generation was obtained using a laminar diffusion flame system by mixing ethylene gas (the primary hydrocarbon fuel) and acetylene gas to compensate for the effect of iron oxide to suppress soot formation. By reaching the vapor phase of iron pentacarbonyl by warming to 20 °C with combusted argon carrier gas (all Sigma-Aldrich Chemical Co., St. Louis, MO) in the presence of an ethylene/acetylene vapor mix, the source of iron was generated. The result of these combusted reactants generated a hetero-disperse aerosol of ultrafine iron oxide particles (Fe_2_O_3_) and associated soot. Further details of the system and particle generation can be found in previously published studies [48, 54, 95]. In order to simulate unhealthy air quality conditions, an average particle concentration of 200 μg/m^3^ was selected, as this reflects a concentration that is reminiscent of heavy pollution and poor air quality days in many parts of the world [48, 97].

### Cardiac perfusion and brain tissue collections

At P15 and P35, one male and one female offspring from each litter were collected from their home cages, anesthetized using isoflurane (2–4% inhalation) and underwent transcardiac perfusion. Briefly, a lateral incision was made in the abdominal wall below the rib cage. With curved scissors, an incision was made in the diaphragm and cuts were made along the ribs to the collarbone to allow the sternum to be lifted. Once exposed, a 15-gauge perfusion needle was inserted into the ascending aorta of the heart for entry of perfusate, and an incision was made into the right atrium to create an outlet for drainage. Using a perfusion pump, 20 mL of PBS was slowly pumped through the circulatory system to reach adequate clearing. Whole brains were removed and dissected into hemispheric halves. One half was further dissected into cortical and hippocampal regions, flash frozen with liquid nitrogen, and stored at − 80 °C for later use in cytokine analyses. The remaining half was placed in 4% PFA for fixation for 24 h. Following this, it was then placed in 30% sucrose for 24 h for cryoprotection. Cryoprotected tissues were then embedded in optimal cutting temperature (O.C.T.) media and frozen at − 80 °C.

### Tissue sectioning and staining

Frozen tissue embedded in O.C.T. was sectioned with a Leica Instruments cryostat at 20 µm. Free-floating tissue sections were stored in PBS containing 0.01% sodium azide. Sections were incubated in 1:1000 rabbit-anti IBA-1 (Wako, Neuss, Germany) with 10% normal goat serum (NGS) and 0.2% triton X-100 at 4 °C for 24 h, followed by 1-h incubation with goat-anti rabbit biotinylated secondary antibody (Vector Laboratories, Burlingame, CA) in 5% NGS for 1 h at room temperature. Tissue sections were then incubated with avidin-biotinylated HRP complex (Vectastain Elite ABC kit, Vector Laboratories, Burlingame, CA) at room temperature. Visualization of labeling was conducted using 3,3’-diaminobenzidine (DAB) solution in the presence of peroxidase (HRP) enzyme. All sections were thoroughly rinsed three times with 1X PBS between staining steps. Sections were mounted onto glass Superfrost Plus microscope slides and cover slipped with VectaMount Permanent Mounting Medium.

### Stereology

IBA-1-positive microglia were identified using stereological methods. IBA-1 cell counts were made on a brightfield microscope (Nikon Eclipse Ci, Nikon, Tokyo) at 20X magnification, and images were taken using NIS Elements v.4.0 (Nikon Instruments Inc. 1300 Walt Whitman Road Melville, NY 11747-3064, U.S.A.). Image analysis was performed using a macros script in ImageJ version 1.53 [98] to remove background noise and isolate IBA-1-positive cells in order to quantify microglia. A total of six to nine sections per brain region per animal were collected. Counts of microglia cells were taken from a 554.7 µm by 1232.1 µm box spanning the infralimbic and anterior cingulate cortical areas of the frontal cortex, as well as the dentate gyrus, CA1, CA2, and CA3 of the hippocampus. Microglia were identified by IBA-1 positive cell body staining. Statistical significance was determined using one-way ANOVA with multiple comparisons on GraphPad Prism version 9.4.1 (GraphPad software, graphpad.com).

### Multiplex bead-based cytokine analysis

Frozen tissues were lysed using cell lysis buffer and total protein from the lysates were quantified via Bradford assay. Analysis of serum cytokines was performed using a multiplex mouse 25-plex bead immunoassay (Milliplex Mouse Cytokine/Chemokine Magnetic Bead Panel #MCYTMAG70PMX25BK). The following cytokines were quantified: granulocyte colony stimulating factor (G-CSF), granulocyte colony stimulating factor (GM-CSF), IFN-γ, IL-1α, IL-1β, IL-2, IL-4, IL-5, IL-6, IL-7, IL-9, IL-10, IL-12 (p40), IL-12 (p70), IL-13, IL-15, IL-17, interferon gamma-induced protein 10 (IP-10), keratinocyte chemoattractant (KC), monocyte chemoattractant protein-1 (MCP-1), macrophage inflammatory protein-1 alpha (MIP-1α), MIP-1β, MIP-2, chemokine ligand 5 (CCL5/RANTES), and tumor necrosis factor alpha (TNF-α). Standards and reagents were all prepared according to the manufacturers’ recommendations. Each lysate was diluted to a standardized concentration of 50 µg and run in duplicate. Twenty-five microliters of sample, standards, or blanks were loaded into a 96-well plate with appropriate amounts of assay buffer and matrix solution. The plate was then incubated overnight with antibody-coupled magnetic beads. The following day, the plate underwent a series of washes. Washes were performed using a Bio-Plex handheld magnet (Bio-Rad Laboratories, Hercules, CA, USA). After the final wash, the plate was incubated with biotinylated detection antibody on a shaker for 30 min, and analyzed using a Bio-Rad Bio-Plex 200 plate reader (Bio-Rad Laboratories, Hercules, CA, USA). The following were the minimal amounts of detectable cytokine concentration: G-CSF: 1.7 pg/mL; GM-CSF: 10.9 pg/mL; IFNγ: 1.1 pg/mL; IL-1α: 10.3 pg/mL; IL-1β: 5.4 pg/mL; IL-2: 1.0 pg/mL; IL-4: 0.4 pg/mL; IL-5: 1.0 pg/mL; IL-6: 1.1 pg/mL; IL-7: 1.4 pg/mL; IL-9: 17.3 pg/mL; IL-10: 2.0 pg/mL; IL-12 (p40): 3.9 pg/mL; IL-12 (p70): 4.8 pg/mL; IL-13: 7.8 pg/mL; IL-15: 7.4 pg/mL; IL-17: 0.5 pg/mL; IP-10: 0.8 pg/mL; KC: 2.3 pg/mL; MCP-1: 6.7 pg/mL; MIP-1α: 7.7 pg/mL; MIP-1β: 11.9 pg/mL; MIP-2: 30.6 pg/mL; RANTES: 2.7 pg/mL; TNF-α: 2.3 pg/mL. Sample concentrations that fell below the minimal detection value were given a proxy value of half the limit of detection for statistical comparisons.

### Statistical analysis

Brain cytokine data were analyzed using linear-mixed effects modeling to control for the unexplained residual variance that could be originating from litter-to-litter variations due to the hierarchical data structure in which statistical independence of observation is violated [12, 61, 62, 107, 122]. Models were built with the lme package of R version 4.2.2 using a forward-stepwise regression approach. First, a random-effects only model was constructed with litter set as the random effect. Then, two fixed effects variables were added for the exposure types: MAA (OVA) and UIS (Soot). A third model was constructed that included both the main effects and the interaction of MAA and UIS followed by a final model containing both treatments, sex, and their interactions. Model fit was assessed using the likelihood ratios test and the best model was selected based on the Akaike Information Criterion (AIC). For models with significant interactions effects, groups were further analyzed using pairwise comparisons of estimated marginal means. Litter size and ratio of male offspring were assessed by two-way analysis of variance (ANOVA).

## Results

### Litter size and sex ratio

Litter sizes averaged 5–6 offspring per litter (see Table 1), with no differences in the number of pups for MAA (*p* = 0.757), UIS (*p* = 0.925), or their interaction (*p* = 0.683). Similarly, no differences were observed between the male-to-female ratio across treatment groups (*p* > 0.05 for all comparisons).

**Table 1:** Litter size and sex ratio

Treatment	Litter size	Ratio of males
PBS/air	5.83 ± 1.83	0.49 ± 0.15
MAA	5.40 ± 1.14	0.34 ± 0.20
UIS	5.50 ± 1.29	0.33 ± 0.13
MAA–UIS	5.60 ± 1.14	0.49 ± 0.23

Ratio of male-to-female offspring was calculated as the number of male mice in a litter divided by the total number of pups in the litter. Litter size and sex ratio data are shown as mean ± SD

### P15 offspring cortex cytokines

**Fig. 1:**
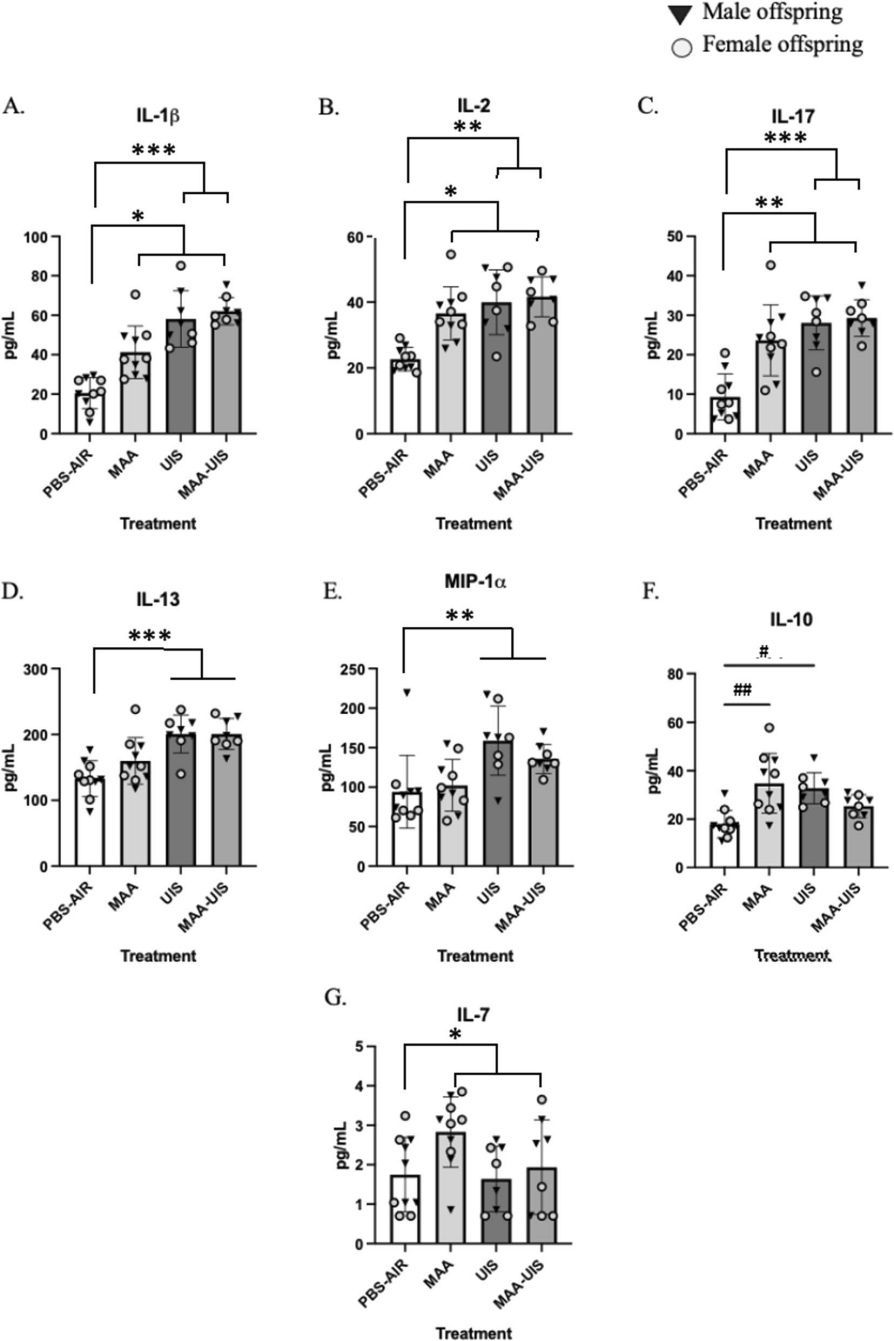
Cortical cytokine concentrations in P15 offspring brains exposed to PBS-AIR, MAA, UIS, or MAAUIS. Cytokines were assessed using multiplex bead-based immunoassays. **A** IL-1β, **B** IL-2, **C** IL-17, **D** IL-13, **E** MIP-1α, **F** IP-10, **G** IL-7 are represented as pg/mL after being normalized to total protein content. Statistical significance was determined by multilevel mixed-effects modeling. Main effects are represented as *p < 0.05, **p < 0.01, ***p < 0.001 compared to PBS-Air; pairwise comparisons are represented as # *p* < 0.05, ## *p* < 0.01

Offspring brains were collected and micro-dissected into cortical and hippocampal sections at P15. Homogenates of each section were then analyzed for cytokine concentration. Multilevel mixed-effects modeling was used to control for within-litter variability and inclusions of fixed effects were determined using forward stepwise regression. For all cytokines measured, the inclusion of sex-difference did not significantly improve model fit (Additional file 1: Table S1). In the cortex, several cytokines were elevated in both male and female offspring of MAA, UIS, and MAA–UIS dams, many of which are generally considered inflammatory and potentially neurotoxic with prolonged exposure (Fig. 1). Among these, three cytokines (IL-1β, IL-2, IL-17) were found to be significantly elevated in all treatment groups. For example, a main effect of exposure for both MAA (*p* = 0.016), and UIS (*p* < 0.001), and MAA–UIS (*p* < 0.001) resulted in an average of a 2- to threefold increase of IL-1β in the cortex, compared to age-matched PBS-AIR controls (Fig. 1). No additional additive effects were observed with the combination of MAA and UIS. Similarly, it was found that there were significant increases in IL-2 for both MAA (*p* = 0.002) and UIS (*p* = 0.001) exposures. For IL-2, the MAA–UIS interaction model identified a significant elevation of IL-2 in the MAA–UIS group compared to the PBS-Air group (*p* = 0.045). However, post hoc analysis did not identify any interaction effects between MAA–UIS and the other treatment conditions. Similarly, a model containing the MAA–UIS interaction for IL-17 confirmed main effects for both MAA (*p* = 0.001) and UIS (*p* < 0.001) exposures, as well as a significant MAA–UIS interaction (*p* = 0.017), but the differences between these treatment conditions were not statistically significant.

The elevation in IL-1β, IL-2, and IL-17 occurred with concomitant increases in the canonical anti-inflammatory marker IL-10. A model containing treatment conditions and their interaction identified effects of both MAA (*p* = 0.001), and UIS (*p* = 0.005) on IL-10 elevations compared to PBS-Air controls. Interestingly, a significant MAA–UIS interaction (*p* = 0.002) identified suppressive effects of the combined MAA–UIS exposure on IL-10 levels. Post hoc analysis confirmed that while the MAA (*p* = 0.007) and UIS (*p* = 0.024) alone both increased the IL-10 concentration compared to PBS-Air, this increase was absent in the MAA–UIS group (*p* = 0.387) and no differences were observed between MAA and UIS conditions (*p* = 0.967).

For several inflammatory markers, only the main effect of UIS significantly altered cytokine concentrations in the cortex. Specifically, the exposure to UIS resulted in an approximately 54 pg/mL (*p* =  < 0.001), and 49 pg/mL (*p* = 0.003) increase in IL-13 and MIP-1α, respectively, compared to the PBS-Air group, and no effect of MAA were observed for either of these two cytokines. Conversely, a main effect of MAA was observed for IL-7 (*p* = 0.050) without an effect of UIS (*p* = 0.167). For both IL-13, MIP-1α, and IL-7, models containing a MAA–UIS interaction were not significant and no sex-by-treatment interactions were identified in any of the P15 cortex cytokine measures.

### P35 offspring cortex cytokines

**Fig. 2:**
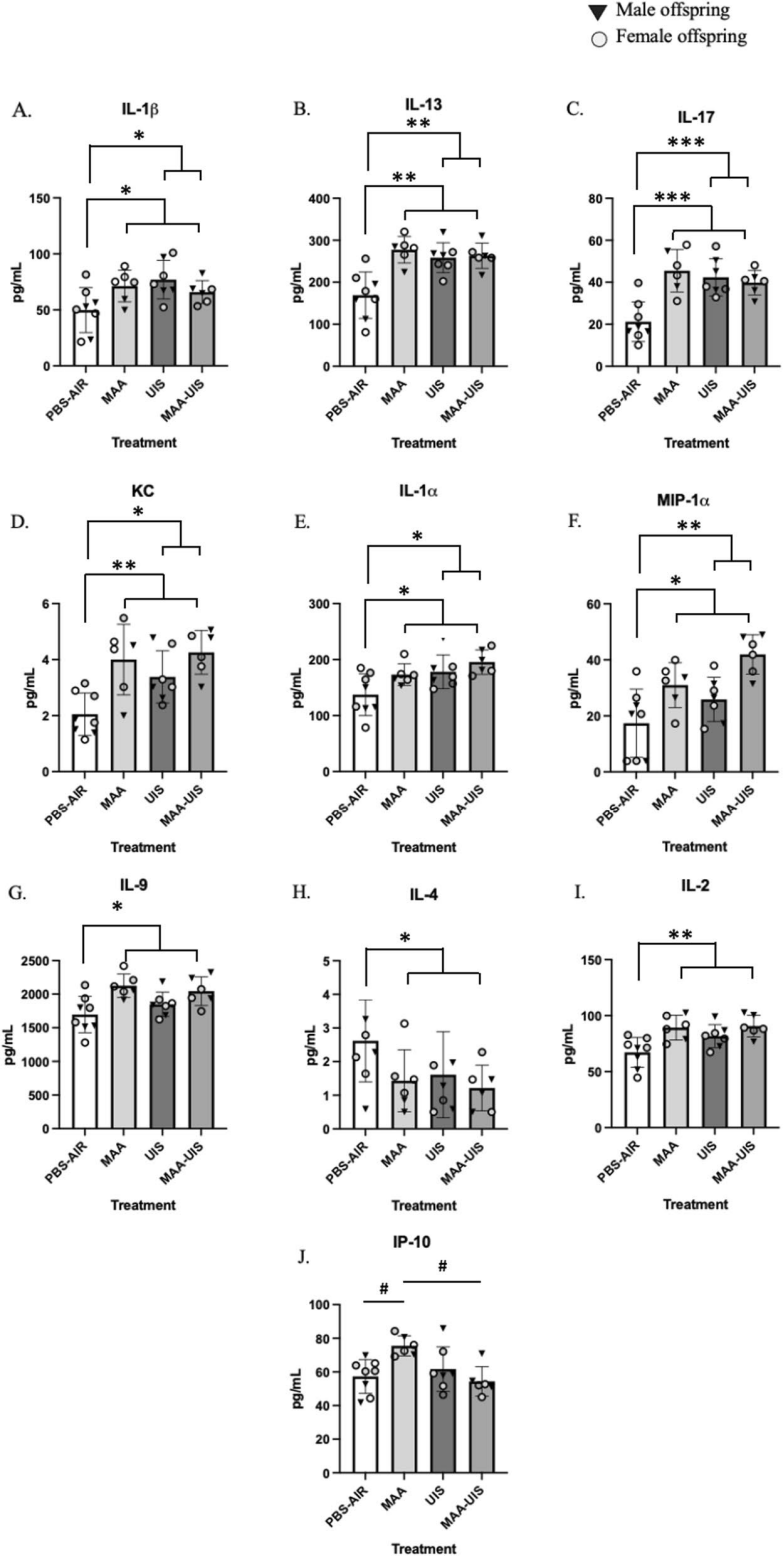
Cortical cytokine concentrations in P35 offspring brains exposed to PBS-AIR, MAA, UIS, or MAAUIS. Cytokines were assessed using multiplex bead-based immunoassays. **A** IL-1β, **B** IL-13, **C** IL-17, **D** KC, **E** IL-1α, **F** MIP-1α, **G** IL-9, **H** IL-4, **I** IL-2, and **J** IP-10 are represented as pg/mL after being normalized to total protein content. Statistical significance was determined by multilevel mixed-effects modeling. Main effects are represented as *p < 0.05, **p < 0.01, ***p < 0.001 compared to PBS-Air

Littermates from the P15 cytokine investigation were left undisturbed until P35, at which time brains were collected and micro-dissected into cortical and hippocampal sections. Mixed-effects models that included sex were not significantly improved over treatment-alone models for all cytokines measured (Additional file 2: Table S2). However, models that included treatment main effect and interaction revealed changes in several cytokines in the cortices of both sexes of MAA, UIS, and MAA–UIS offspring at P35 in a similar manner to what was identified in P15 offspring (Fig. 2). Many of the cytokine changes observed at P15 remained altered in the P35 offspring cortex. For instance, it was revealed that there were effects of MAA and UIS in IL-1β, IL-13 and IL-17 cortex of P35 offspring compared to PBS-AIR control mice (IL-1β: MAA *p* = 0.046, UIS *p* = 0.008; IL-13: MAA *p* = 0.002, UIS *p* = 0.005; IL-17: MAA *p* < 0.001, UIS *p* = 0.001) (see Additional file 2: Table S2). While mixed-effects models were selected that included an MAA–UIS interaction for these cytokines, post hoc analysis revealed no additive or suppressive effects of the combined exposure of MAA and UIS. Elevations in IL-1β, IL-13, and IL-17 were similar between both MAA- and UIS-alone groups (*p* > 0.05 for all comparison). Similarly, main effects for both MAA and UIS exposure were observed in the P35 cortex for KC, IL- α, and MIP-1α (KC: MAA *p* = 0.002, UIS *p* = 0.050; IL-1α: MAA *p* = 0.050, UIS *p* = 0.013; MIP-1α: MAA *p* = 0.002, UIS *p* = 0.021). No differences were observed in any of these cytokine concentrations between in the three exposure groups and no interactions for MAA–UIS were observed in the mixed effects models.

For several cytokines the individual contribution of MAA, but not UIS, impacted specific inflammatory profiles. Specifically, MAA treatment resulted in an increase in IL-9 (*p* = 0.012), IL-4 (*p* = 0.037), and IL-2 (*p* = 0.005) compared to offspring born from PBS-Air exposed dams. Of all the cytokines investigated, IP-10 concentration was the only identified cytokine where a combination of MAA and UIS resulted in suppressive effects. Specifically, the mixed-effects model revealed that MAA exposure alone significantly increased the level of IP-10 in the P35 cortex compared to PBS-Air offspring, with an additional interaction identified in the combination of MAA and UIS for IP-10 (*p* = 0.008) suggesting a suppressive effect by UIS. Post hoc analysis confirmed that MAA-only offspring had significantly higher levels of IP-10 compared to MAA–UIS mice (*p* = 0.026), and no differences were observed between the MAA–UIS condition and PBS-Air control mice (*p* = 0.977).

### P15 offspring hippocampus cytokines

**Fig. 3:**
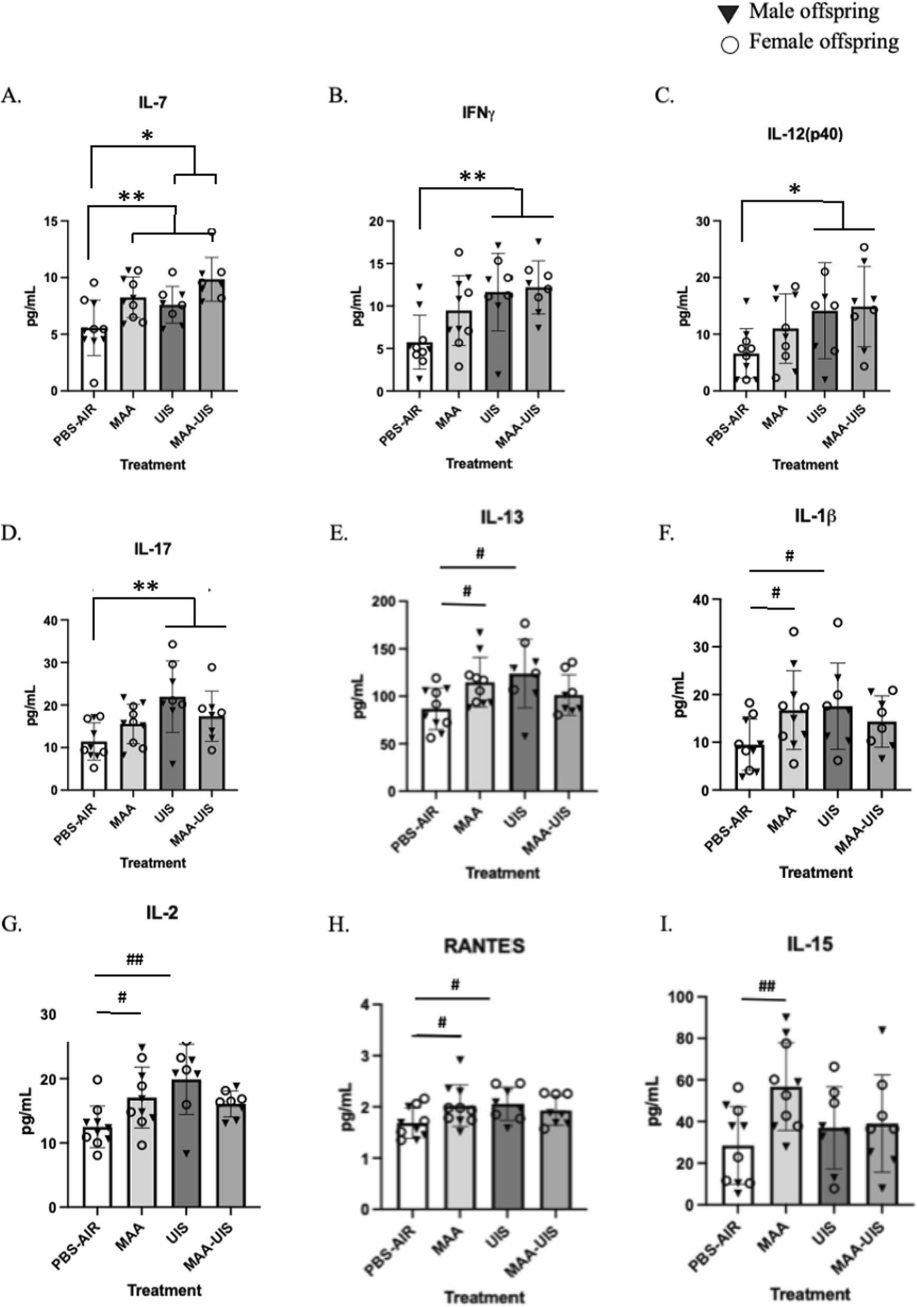
Hippocampal cytokine concentrations in P15 offspring brains exposed to PBS-AIR, MAA, UIS, or MAAUIS. Cytokines were assessed using multiplex bead-based immunoassays. **A** IL-7, **B** IFNγ, **C** IL-12(p40), **D** IL-17, **E** IL-13, **F** IL-1β, **G** IL-2, **H** RANTES, and **I** IL-15 are represented as pg/mL after being normalized to total protein content. Statistical significance was determined by multilevel mixed-effects modeling. Main effects are represented as *p < 0.05, **p < 0.01, compared to PBS-Air; pairwise comparisons are represented as #*p* < 0.05, ##*p* < 0.01

Along with cortical sections, hippocampal homogenates from the same offspring brains were analyzed at P15 for cytokine concentrations. For all cytokines measured, the inclusion of sex in multi-level models did not significantly improve model fit (Additional file 3: Table S3). Similar to what was uncovered in the respective cortical sections, several inflammatory cytokines were elevated in male and female offspring of MAA, UIS, and MAA–UIS dams (Fig. 3). Interestingly, main effects for both MAA (*p* = 0.002) and UIS (P = 0.005) were only observed for IL-7 in the P15 hippocampus. None of the other cytokines investigated reached statistical significance in all three treatment groups compared to controls. However, IFNγ concentrations showed a main effect for UIS (*p* = 0.007) but not MAA (*p* = 0.111), and similar elevations were observed in IL-12(p40) and IL-17 for UIS alone or with MAA–UIS (IL-12(p40): *p* < 0.007; IL-17: *p* < 0.002). These increases were not present in the hippocampus of offspring born from the MAA treatment-alone group. Interestingly, many cytokines measured in the P15 hippocampus showed suppressive effects of the combined treatment of MAA and UIS. For example, a mixed-effects model for IL-13 containing a treatment interaction revealed a significant effect of MAA-alone (*p* = 0.034) and UIS-alone (*p* = 0.011) as well as a negative effect of the MAA–UIS interaction (*p* = 0.014). Post hoc comparisons confirmed that the IL-13 concentrations in the MAA–UIS offspring hippocampus were no different from PBS-Air mice (*p* = 0.272). However, differences between MAA-alone or UIS-alone groups were also not statistically different from the MAA–UIS condition (*p* > 0.05 for all comparisons. Similarly, these intermediate effects were also observed for IL-1β (MAA *p* = 0.047, UIS *p* = 0.038), IL-2 (MAA *p* = 0.026, UIS *p* = 0.002), and RANTES (MAA *p* = 0.041, UIS *p* = 0.034). Finally, one additional cytokines was found to be impacted by MAA alone compared to the control, and not the UIS or MAA–UIS treatment groups. Specifically, IL-15 was found to be elevated by MAA (*p* = 0.009), but not UIS or MAA–UIS.

### P35 offspring hippocampus cytokines

Hippocampal homogenates from P35 offspring were also assessed for cytokine differences; however, multi-level mixed-effects modeling did not reveal any significant effects of treatment or sex in on the concentration of all cytokines measured (*p* > 0.05; see Additional files 1, 2, 3, 4).

### P15 Microglia density

**Fig. 4:**
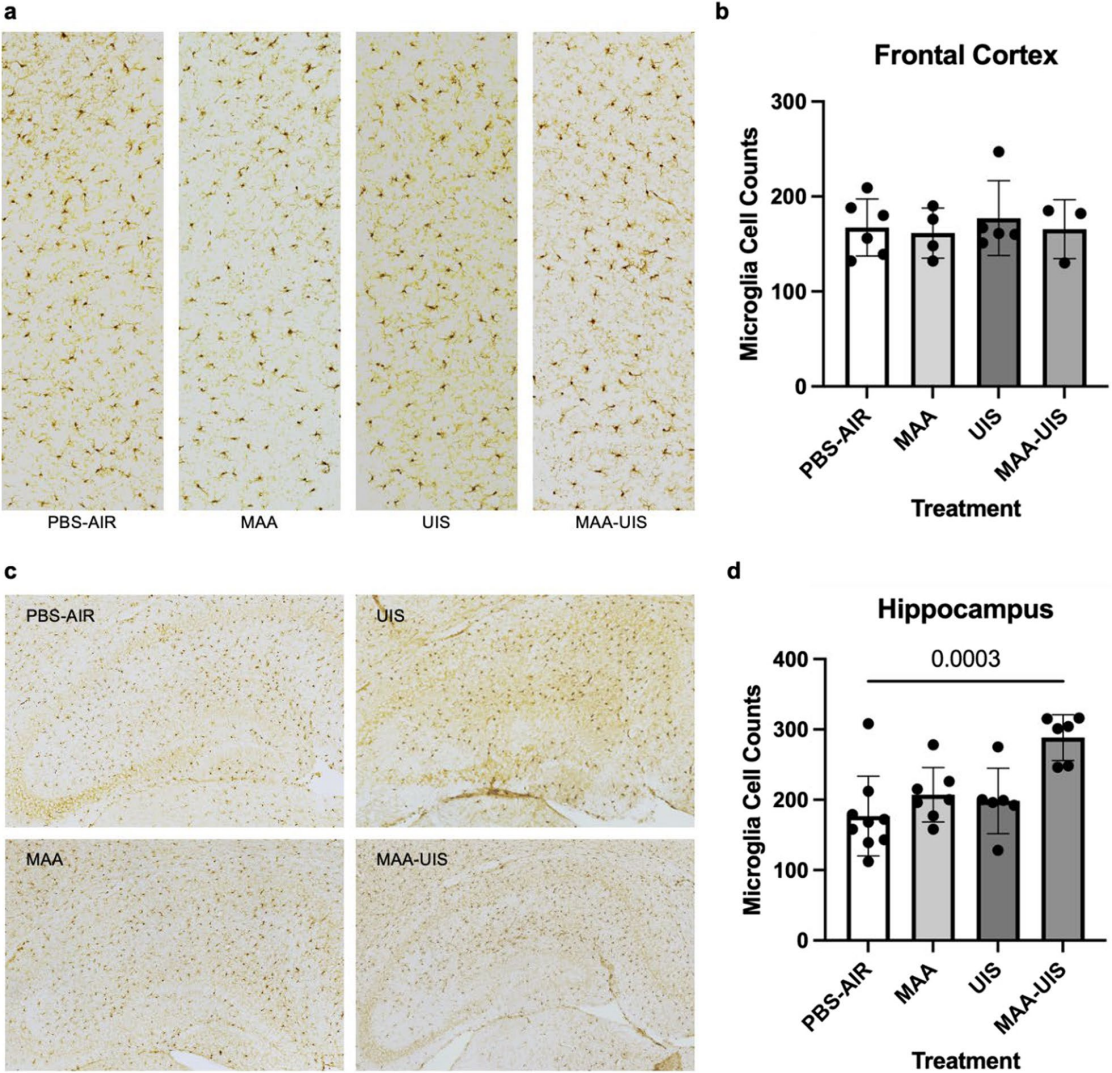
Microglia density in the hippocampus and frontal cortex of MAA, UIS, and MAA-UIS of P15 offspring. **a** and **c** Staining for IBA-1 with diaminobenzidine (DAB) was used to label microglia in coronal sections of p15 mice in the (**a**) frontal cortex and (**c**) hippocampus. **b** and **d** Quantification of microglial density in the (**b**) frontal cortex and (**d**) hippocampus. Statistical significance determined via one-way ANOVA. In the frontal cortex, *n* = 6 (PBS-AIR), *n* = 4 (MAA), *n* = 5 (UIS), *n* = 3 (MAA-UIS). In the hippocampus, *n* = 9 (PBS-AIR), *n* = 7 (MAA), *n* = 6 (UIS), *n* = 6 (MAA-UIS)

Microglia within a 554.7 µm by 1232.1 µm box spanning the anterior cingulate cortex, prelimbic area, and infralimbic area were counted using ImageJ by an investigator blinded to treatment condition. Analysis of microglia density in the dentate gyrus, CA1, CA2, and CA3 regions of the hippocampus in P15 mice showed significant differences between the PBS-air group and the MAA–UIS group (*p* = 0.0166; Fig. 4). There were no significant changes in the microglia density between treatment groups in the frontal cortex of P15 mice (Fig. 4). Additionally, in both the hippocampus and cortex, no differences in microglia density were seen in the single treatment groups (MAA or UIS) when compared to the PBS-air group.

## Discussion

Among the many well established environmental factors that can impact fetal neurodevelopment, asthma and air pollution represent two major sources of immune stimulation that are on the rise, making them a significant concern for pregnant individuals. Based on previous studies of maternal immune activation with asthma, and PM exposure during pregnancy, we hypothesized that the combination of these two environmental stimuli would result in an exacerbated neuroimmune response in offspring. Although the appearance of an additive or synergistic effect of MAA and UIS exposure combined was limited, we did identify increases in cytokine concentrations across all treatment groups in the cortex and hippocampus that may suggest converging pathways for each insult/exposure. However, we also observed potentially suppressive activities that may suggest activation of competing or diverging pathways. Importantly, some of changes appear to be sustained across treatment groups from adolescence into early adulthood in the cortex, demonstrating lasting impacts of these gestational exposures on the neuroimmune environment later in life. Although we identified increases in cytokines in the hippocampus within all treatment groups at P15, these did not remain elevated into early adulthood. Overall, these data show that MAA and UIS environmental stimuli can result in an altered neuroimmune environment that persists from juvenile to adult timepoints.

The allergic response in the lungs of individuals with asthma is characterized by an influx of immune cells, such as neutrophils, mast cells, macrophages, and T-helper (T_H_)2 cells. Our mouse model of MAA previously showed elevated IL-4, IL-5, IL-17, and IFNγ in the lung and peripheral blood of mice exposed to aerosolized OVA during pregnancy [20, 110, 115]. These increases in maternal serum cytokines result in neuro-immune signaling changes in the fetal brain during in utero development [115]. Our present data extend these findings by revealing evidence of increases in cortical and hippocampal cytokines in juvenile mice of MAA dams. In the cortex, for example, MAA increased IL-1β, IL-2, IL-17, IL-10, and IL-7. In the hippocampus, we identified IL-1β, IL-2, IL-7, IL-13, IL-15, and RANTES as being elevated in P15 offspring of MAA-exposed dams. These observed increases in MAA compared to PBS-AIR controls demonstrate the independent neurodevelopmental impact of allergic inflammation during pregnancy on offspring neuroinflammation. In addition to these findings in the MAA-only treatment group, we also identified suppressive effects of combining MAA with UIS in the cortex in juvenile offspring, notably, IL-10 an anti-inflammatory cytokine, whose suppression may suggest a lack of immune regulation and shift towards more neuroinflammation. Moreover, increases in cytokines as a result of UIS exposure alone were also identified in the hippocampus of juvenile offspring, with elevated IFNγ, IL-1β, IL-7, and IL-12(p40). To the authors’ knowledge, investigations into these neurobiological outcomes in offspring under UIS exposure during gestation have not been previously reported, highlighting the novelty of our model and findings.

In addition to independent effects of MAA or UIS treatment on cytokine concentrations in the cortex and hippocampus of juvenile offspring, several cytokines did not respond further to the combined effects of MAA and UIS. Most notably, we observed similar elevations in the cortex of IL-1ɑ, IL-1β, IL-2, and IL-17 in dual-exposed MAA–UIS offspring suggesting a potential ceiling effect produced by either environmental insult alone, or a maximal response through converging signaling pathways. IL-1β, IL-13, and IL-17 were also trending or increased in the MAA and UIS single treatment groups as well as the MAA–UIS treatment group at P15, and they remained elevated into the P35 timepoint in the cortex. Our data suggest a sustained elevation in these cytokines from P15 to P35 as a result of both MAA and UIS that have the potential for long-lasting impacts on neurodevelopment in the cortex of these offspring.

Consistent with the pleiotropic nature of cytokines in the central nervous system (CNS), IL-1β, IL-2, and IL-17 have all been identified as having neurotrophic properties [5, 13, 29, 58, 66, 69, 78, 92] (Rochman et al. 2013). Indeed, high concentrations (500 ng/mL) of IL-1β can have neurotoxic effects on neurons when exposed for 3–5 days [92], and IL-17 is detected at high levels in the CNS in multiple sclerosis and associated with the neuroinflammatory pathology of the disease [58, 66]. Compared to these neurotoxic concentrations, our data represent moderate increases in cytokines with less than 2.5-fold increases in treatment groups compared to PBS-AIR controls, and may not represent overt inflammation per se. However, these smaller changes in brain cytokine levels during the juvenile period may be biologically significant given their alternative functions in promoting neuronal survival and neurodevelopment. For example, IL-1β acts as a chemokine that guides neurite outgrowth in cortical neurons [69], and IL-17 acts in initiating the release of brain-derived neurotrophic factor (BDNF), glia-derived neurotrophic factor (GDNF), and nerve growth factors (NGF) that are associated with neuronal cell survival and repair [78]. Taken from this view, these cytokines, which are generally considered overtly inflammatory, may be having a more subtle impact on the neuroarchitecture of offspring brains than models finding dramatic increases in concentration of IL-1β and IL-17.

Further demonstrating the neuropoietic nature of these cytokines within the CNS and adding to the idea that the moderate increases observed in this model may be altering the neuropatterning of the offspring brains, IL-2 has been found to have neurotrophic properties and is necessary for proper cytoarchitecture in development [8, 8, 9, 9]. Both IL-2 and IL-13 are among several cytokines that are known to decrease in concentration at P14 under homeostatic conditions, and this developmental timepoint in mice is characterized by a high degree of synaptogenesis and pruning [37, 81]. In contrast, our model, which investigated cytokine concentrations at P15, still within this window of high synaptogenesis, showed increased IL-2, representing a shift in homeostatic load. Taken together, it may be that these sustained moderate increases in cytokines of the cortex are changing the trajectory of cortical development and promoting altered connectivity in the cortex linked to behavioral changes such as decreased social interaction and repetitive behaviors previously identified in our model [19, 109]. This phenomenon of altered connectivity has also been implicated in the core behaviors associated with ASD, specifically the social deficits and restrictive and repetitive behaviors [24, 73, 77].

Similar to our findings in the cortex, we also identified elevations in several cytokines, most notably IFNγ and IL-7, at P15 in the hippocampus of MAA and/or UIS groups compared to control offspring. IFNγ receptors are present on both neurons and glia [90]. In the hippocampus, IFNγ appears to play a role in synaptogenesis [65]. Some investigators have found that overexpression resulted in increased neurogenesis in the dentate gyrus, and because of its neuromodulatory effects, it has been suggested that this may impact cognition and social behavior as a result [7, 35, 36]. Additionally, IL-7 promotes survival and neurite outgrowth in hippocampal neurons [70, 76]. Considering the effects of IL-7 and IFNγ, and that the hippocampus is a major neurogenic niche in the developing brain, future studies may benefit from investigating the potential for hippocampal overgrowth in offspring brains in response to UIS or allergic asthma inflammation during pregnancy. Indeed, this phenomenon of hippocampal overgrowth has been identified in cases of ASD [43, 86, 104] and has been implicated in the deficits associated with emotion perception and sensory processing in ASD individuals [6, 43]. Curiously, the observed increases in hippocampal cytokine concentrations at P15 were not observed in any treatment group of P35 offspring compared to PBS-AIR controls. Although we can only speculate about these findings, it may be that these changes resolve during adolescence when additional brain maturation may be present to compensate for developmental overgrowth much in the same way that volumetric increases in the hippocampus of ASD individuals do not persist into adulthood [43, 104]. Although IL-7 and IFNγ are only two examples of cytokines that were elevated across the treatment groups, they illustrate the broader findings that treatment with MAA, UIS, or MAA–UIS can alter the hippocampal neuroimmune environment.

Prenatal insults have been shown to have lifelong impacts on microglia function and are suspected to play a prominent role in NDD [32, 38, 50, 59, 112, 117]. In ASD, some postmortem studies have identified differences in microglia density and morphology in brains of individuals [60, 82, 83]. In our previous study of MAA, we found DNA methylation differences in adult microglia, and several of these changes occurred in regulatory genes that are shared among some ASD individuals [117]. Given these findings, we sought to examine the density of microglia in the P15 brains of our MAA and UIS exposure model. We observed a significant increase in microglia density within the hippocampus of offspring exposed to MAA–UIS, but these increases were not present in the frontal cortex. One plausible explanation for why these increases were only observed in the hippocampus may be due to the higher density of microglia known to be present in the hippocampus. This higher density of microglia is thought to make the hippocampus more vulnerable to inflammation [18, 101], and disruptions in the dentate gyrus have been linked to NDD [15, 124]. Our findings of increased microglia density in the hippocampus of MAA–UIS offspring mirror data from another maternal immune activation model that utilizes the viral mimic poly(I:C). Specifically, Juckel et al. [56] reported an increase in microglia density in the hippocampus but not the cortex of offspring born from immune-activated dams. Similarly, another study of maternal immune activation using LPS stimulation showed an increase in microglia density in the hippocampus [30]. While it is difficult to make conclusions about microglial function based on density data alone, our observed difference in the hippocampus in combination with similar reports from other maternal immune activation models [30, 56], suggest that asthma allergy and PM mediated immune activation during pregnancy can result in a deviation from homeostatic activity in the offspring hippocampus. Of interest, IFNγ and IL-12 were increased in MIA-UIS in the hippocampus, cytokines that are often associated with a T_H_1 response and M1 macrophage/microglia skewing. These cytokines were not elevated in the cortex. Moreover, in the cortex and hippocampus UIS is associated with increased IL-13 that may balance the signals from IL-12 and IFNγ as seen in peripheral responses. IL-13 is often considered anti-inflammatory in the CNS, with some studies pointing to a neuroprotective impact in CNS diseases and injuries [45, 63, 75, 91]. Although speculative, the results may suggest combined inflammatory M1/T_H_1 associated cytokines IFNγ and IL-12, could influence the MAA–UIS microglia density results while these are protected in the cortex or under UIS conditions through IL-13 production. More investigation is needed on the combination of cytokines released and action on microglia cells in the context of environmental exposures.

Although we did not collect maternal data in this preliminary study, data from previous MAA studies demonstrate increased systemic inflammation characteristic of an allergic asthma response, specifically with increased IL-4, IL-5, and IL-13 [19, 110, 115], suggesting the potential for a similar response in dams of MAA in the current model. Speculation about the systemic impacts of UIS on the maternal immune system, however, is difficult. Many studies of PM exposure suggest IL-6, IL-8, and TNFα as the main cytokines upregulated in response to PM exposure [41, 67, 79, 89, 111]. This difference in cytokine response highlights the potential reason we see differences in the impact between MAA and UIS in our model. However, models of PM can vary widely in the size of PM and composition [79], making speculation about the maternal response in the UIS groups, and the potential role this plays in offspring neurodevelopment, difficult. This variation in PM studies underscores the need for future investigations to identify the maternal cytokine milieu in this model.

While our findings do not necessarily demonstrate an additive effect of MAA and UIS with regard to the cytokines we investigated, we did see a synergistic impact of MAA–UIS on microglia density in the hippocampus. These findings demonstrate the potential for additive effects of maternal asthma exposure when coupled with PM exposure. Independently, studies have shown in both humans and animal models that PM exposure during pregnancy can increase the susceptibility of offspring developing asthma [49, 84, 123]. This increased susceptibility of asthma in offspring is also seen in children of asthmatic mothers [71, 85] (Mattes et al. 2013), suggesting the potential for systemic immune dysfunction when these two stimuli are combined during pregnancy. The findings in this unique model of MAA and UIS exposure highlight the importance of investigating the impact of these closely linked and prevalent environmental factors.

## Conclusions

Our data add to our previous studies on the impact of MAA on fetal brain development, showing here that this model impacts region-specific cytokine concentrations in both the juvenile and adolescent periods. To the investigators’ knowledge, this was the first study to assess the impact of ultrafine iron-soot exposure during gestation on offspring neurobiology. Taken together, these data highlight the importance of understanding the impact that common environmental stimuli can have on fetal development, and the potential for these stimuli to have long-lasting changes in offspring.

### Supplementary Information

**Additional file 1.** Table S1.

**Additional file 2.** Table S2.

**Additional file 3.** Table S3.

**Additional file 4.** Table S4.

## Data Availability

The datasets used and/or analyzed during the current study are available from the corresponding author on reasonable request.

## Abbreviations

ASD: Autism spectrum disorders
PM: Particulate matter
MAA: Maternal allergic asthma
UIS: Ultrafine iron-soot
OVA: Ovalbumin
GD2: Gestational day 2
PBS: Phosphate buffered saline
P: Postnatal days
IL: Interleukin
IFNγ: Interferon gamma
MAA–UIS: MAA and UIS exposure
LPS: Lipopolysaccharide
Poly(I:C): Polyinosinic:polycytidylic acid
ADHD: Attention deficit, hyperactive disorder
IS: Iron-soot
NGS: Normal goat serum
DAB: 3,3’-Diaminobenzidine
G-CSF: Granulocyte colony stimulating factor
GM-CSF: Granulocyte colony stimulating factor
IP-10: Interferon gamma-induced protein 10
KC: Keratinocyte chemoattractant
MCP-1: Monocyte chemoattractant protein-1
MIP-1α: Macrophage inflammatory protein-1 alpha
RANTES: Chemokine ligand 5
TNF-α: Tumor necrosis factor alpha
AIC: Akaike information criterion
T_H_: T-helper
CNS: Central nervous system
BDNF: Brain-derived neurotrophic factor
GDNF: Glia-derived neurotrophic factor
NGF: Nerve growth factors
MIA: Maternal immune activation
