# Supplementary material for: Characterizing the neuroimmune environment of offspring in a novel model of maternal allergic asthma and particulate matter exposure
Source: J Neuroinflammation. 2023 Nov 2;20:252. doi: 10.1186/s12974-023-02930-7 (PMC10621097; doi:10.1186/s12974-023-02930-7)
Supplement: Supplementary file 1 — Additional file 1. Table S1. [file 12974_2023_2930_MOESM1_ESM.docx]

G-CSF

INFy

IL-1a

IL-1b

IL-2

IL-4

IL-7

IL-9

IL-10

IL-12p40

IL-13

IL-15

IL-17

IP-10

MCP-1

MIP-1a

MIP-2

RANTES
