# Supplementary material for: Characterizing the neuroimmune environment of offspring in a novel model of maternal allergic asthma and particulate matter exposure
Source: J Neuroinflammation. 2023 Nov 2;20:252. doi: 10.1186/s12974-023-02930-7 (PMC10621097; doi:10.1186/s12974-023-02930-7)
Supplement: Supplementary file 4 — Additional file 4. Table S4. [file 12974_2023_2930_MOESM4_ESM.docx]

**P35 Hippocampus**

G-CSF

GM-CSF

IFNg

IL-1a

IL-1b

IL-2

IL-4

IL-5

IL-6

IL-7

IL-9

IL-10

IL-12p40

IL-12p70

IL-13

IL-15

IL-17

IP-10

KC

MCP-1

MIP-1a

MIP-1b

MIP-2

TNFa
